# Petechial and subcutaneous hemorrhage with blister formations in right upper extremity during cardiopulmonary bypass by axillary artery cannulation

**DOI:** 10.1186/s40981-015-0008-3

**Published:** 2015-08-27

**Authors:** Kei Kamiutsuri

**Affiliations:** Department of Anesthesiology, Rinku General Hospital, Rinku Ourai Kita 2-23, Izumisanoshi, Osaka 598-8577 Japan

**Keywords:** Axillary artery cannulation, Cardiopulmonary bypass, Acute compartment syndrome

## Abstract

A 72-year-old man underwent aortic valve replacement and coronary artery bypass graft using cardiopulmonary bypass with right axillary artery cannulation. After undraping, petechial and subcutaneous hemorrhage with blister formations were found in right upper extremity. Axillary artery cannula was considered to compress right subclavian and disturb venous return in the right subclavian vein, which caused an acute compartment syndrome during cardiopulmary bypass. This case was a rare,but severe complication of cardiopulmonary bypass with right axillary artery cannulation.

## Correspondence/findings

**Fig. 1:**
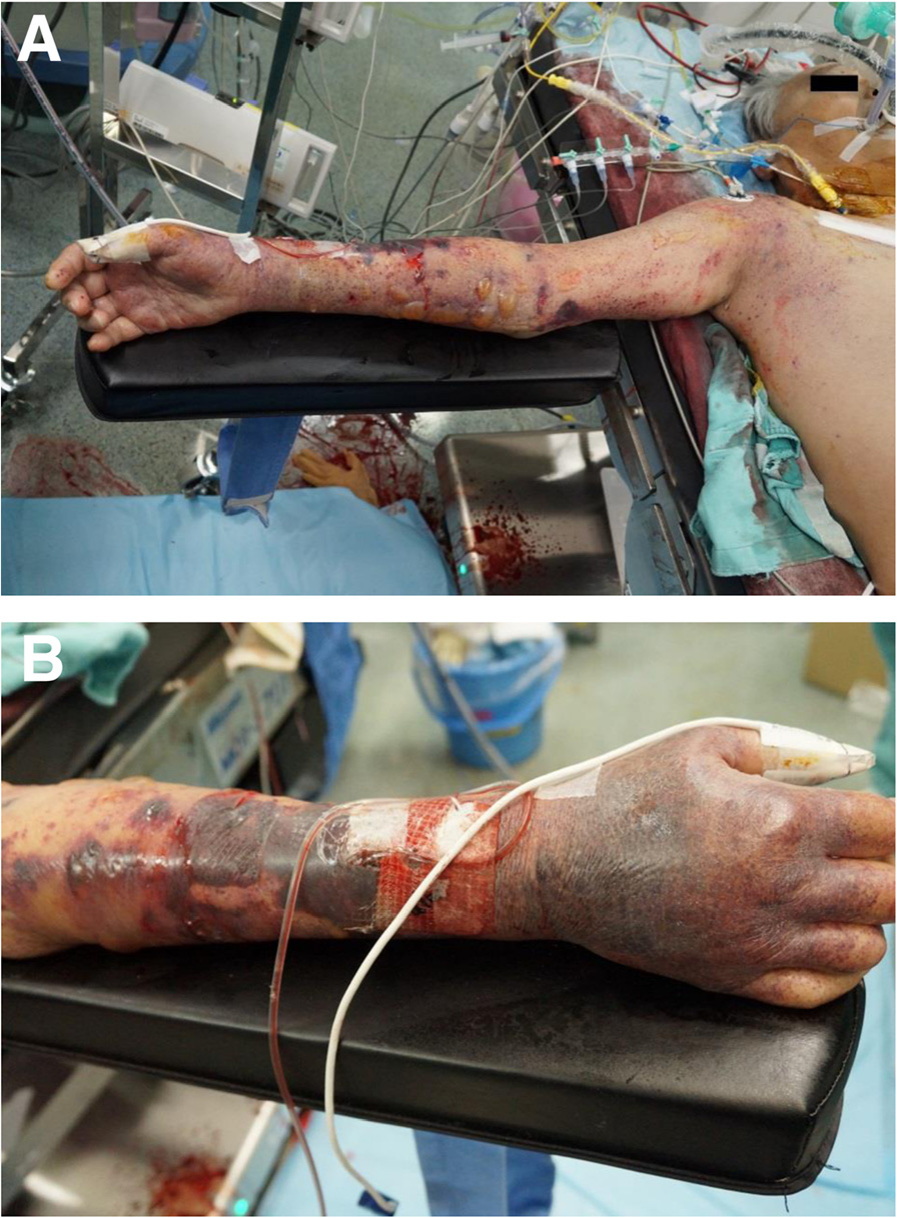
**a** Petechial and subcutaneous hemorrhage with blister formations in right upper extremity. **b** Subcutaneous hemorrhage was most severe around the cannulation site of peripheral venous catheter

A 72-year-old man underwent aortic valve replacement and coronary artery bypass graft. He was a maintenance hemodialysis patient and had a dialysis arterial venous shunt in the left forearm. Peripheral venous catheter 24G was already placed in the right forearm, and central venous catheter and pulmonary artery catheter were inserted in the right jugular vein before induction of anesthesia. Noninvasive blood pressure cuff was not used since arterial line was already placed in left femoral artery. Most intraoperative transfusion or agents were given via central venous catheter. Cefazolin 1 g with 100 ml of normal saline and about 300 ml of normal saline were given via peripheral venous catheter during operation, and intravenous fluid via peripheral venous catheter was stopped after induction of cardiopulmonary bypass. Cardiopulmonary bypass with right axillary artery cannulation bypass was established due to severe calcification of ascending aorta. The waveform and saturation of pulse oximeter in the right thumb were normally displayed after weaning from cardiopulmonary bypass. When drapes on patient were removed, petechial and subcutaneous hemorrhage with blister formations were found in right upper extremity (Fig. 1a) and subcutaneous hemorrhage was most severe around the cannulation site of peripheral venous catheter (Fig. 1b). Acute compartment syndrome was diagnosed from these findings.

Acute compartment syndrome during cardiopulmonary bypass with axillary artery cannulation are a few and rare complication [1]. In this case, axillary artery cannulation itself might compress the right subclavian vein, and acute compartment syndrome result from transient disorder of venous return. Subcutaneous hemorrhage was considered to be exacerbated by leakage of venous blood extended through insertion site due to elevated venous pressure.

In conclusion, acute compartment syndrome is one of complications for cardiopulmonary bypass with axillary artery cannulation, and the peripheral venous catheter should not be placed on the side of axillary artery cannulation.
